# The Role of Interleukins in HBV Infection: A Narrative Review

**DOI:** 10.3390/jpm13121675

**Published:** 2023-11-30

**Authors:** Konstantinos Dimitriadis, Stamatia Katelani, Maria Pappa, George E. Fragkoulis, Theodoros Androutsakos

**Affiliations:** 1 Department of Pathophysiology, Medical School, National and Kapodistrian University of Athens, 11527 Athens, Greece; kdimitriadis@med.uoa.gr (K.D.); stamkatelani@med.uoa.gr (S.K.); 2 First Department of Internal Medicine, Propaedeutic Clinic, “Laiko” Hospital, National and Kapodistrian University of Athens, 11527 Athens, Greece; mariakpappa@med.uoa.gr (M.P.); geofragkoul@med.uoa.gr (G.E.F.); 3 Institute of Infection, Immunity and Inflammation, University of Glasgow, Glasgow G12 8QQ, UK

**Keywords:** Hepatitis B virus, interleukins, chronic liver disease, immune response

## Abstract

Hepatitis B virus (HBV) infection is a worldwide medical issue with significant morbidity and mortality, as it is the main cause of chronic liver disease and hepatocellular carcinoma (HCC). Both innate and adaptive immune responses play a key role in HBV replication and suppression. Recently, the pathophysiological function of interleukins (IL) in the natural course of HBV has gained much attention as a result of the broad use of anti-interleukin agents for a variety of autoimmune diseases and the accompanying risk of HBV reactivation. We present a narrative review regarding the role of IL in HBV infection. Collectively, the pro-inflammatory ILs, namely IL-1, IL-5, IL-6, IL-12 and IL-21, seem to play a critical role in the suppression of HBV replication. In contrast, the anti-inflammatory cytokines IL-10, IL-23 and IL-35 probably act as HBV replication enhancers, while IL-17 has been correlated with HBV-related liver injury. Interestingly enough, IL-2, IL-4 and IL-12 have been tried as therapeutic options against HBV infection with contradictory results. Lastly, the role of IL-22 remains largely ill defined, although preliminary data suggest that it may play a significant role in HBV replication, proliferation and subsequent liver damage.

## 1. Introduction

Hepatitis B virus (HBV) infection is a worldwide healthcare issue with significant morbidity and mortality, and it is the primary cause of chronic liver disease and hepatocellular carcinoma (HCC) [1,2]. According to the World Health Organization (WHO), in 2019, 296 million people worldwide were suffering from chronic HBV infection (CHB), with 1.5 million new infections each year, while the HBV burden was estimated at 116 million people in Western Pacific regions, 81 million people in Africa and 14 million people in Europe; in the same year, hepatitis B resulted in approximately 820,000 deaths [3].

HBV primarily affects hepatocytes and replicates within them. The virus enters the hepatocyte in 2 ways: via a low-affinity binding reaction between hepatitis B surface antigen (HBsAg) and the heparan sulfate proteoglycans (HSPG) on the cell surface and via a high-affinity binding reaction between the N-terminal part of the pre-S1 region of HBsAg and the hepatic bile acid transporter sodium taurocholate co-transporting polypeptide (NTCP), the main HBV hepatocyte receptor [4]. After entering the hepatocyte, relaxed circular DNA (rcDNA) is transported into the nucleus and converted into covalently closed circular DNA (cccDNA) [5]. In this way, HBV DNA remains in the hepatocyte, even after successful treatment with nucleos(t)ide analogues. In both chronic and acute infections, HBV induces liver injury mainly in a non-cytopathic way, mediated by the activation of the immune system.

Coordinated innate and adaptive immune responses are important for effective HBV clearance during acute infection. However, when the immune system fails to mount an effective response, viral persistence and consequent CHB occurs.

**Figure 1:**
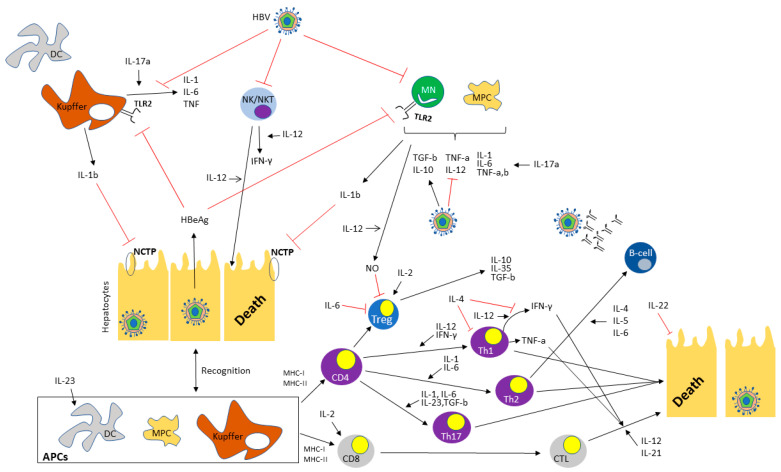
The complex interplay between different cytokines and hepatitis B virus. Black arrows indicate enhancement or secretion. Red arrows indicate suppression. Abbreviations: HBV: Hepatitis B virus, DC: dendritic cell, IL: Interleukin, NK: Natural killer, NKT: Natural killer T-cell, TLR2: Toll-like receptor 2, TNF: Tumor necrosis factor, IFN-γ: Interferon-gamma, TGF-b: Transforming growth factor beta, HbeAg: Hepatitis B envelope antigen, NTCP: Sodium taurocholate co-transporting polypeptide, NO: Nitric oxide, MHC: Major histocompatibility complex, Tregs: Regulatory T-cells, Th1-cells: T helper cells type 1, Th2-cells: T helper cells type 2, Th17-cells: T helper cells type 17, APCs: Antigen presenting cells, CTL: Cytotoxic T-cell.

Interleukins (ILs), as fundamental elements of the immune system, regulate the outcome and characteristics of the adaptive immune response in a key manner. They play a crucial role in cellular communication by being generated and exerting their effects in a wide array of cell types; in this light, they form an intricate regulatory network. Up until now, 40 different ILs have been discovered, having 3 main functions, namely activating and regulating immune cells, transmitting information in a variety of cells, and participating in inflammatory response [6]. Regarding HBV infection, ILs seem to play an important role in viral persistence as well as in continuous liver damage (Table 1 and Figure 1), a role that has gained attention after the wide use of anti-IL agents for a variety of autoimmune diseases.

Herein, we discuss the role of each IL in the pathophysiology of HBV infection and present the available data (Table 1 and Figure 1).

## 2. Interleukin 1

Interleukin-1a (IL-1a) and interleukin-1b (IL-1b) belong to pro-inflammatory cytokines, with local and systemic roles, respectively [7]. When IL-1 binds to its receptor (IL-1R), it induces the production of other pro-inflammatory cytokines, like interleukin-6 (IL-6) [8].

IL-1 seems to inhibit HBV entry in the hepatocyte by down-regulating the expression of NTCP and blocking cccDNA transcription in 2 different ways: (a) through the inhibitory binding of nuclear factor-kB (NF-kB) to cccDNA and (b) through hepatocytes’ de-differentiation. The latter leads to loss of hepatocyte nuclear factor-4a (HNF4a), a transcription factor controlling HBV gene expression and replication, and thus to suppression of cccDNA transcription [8].

Experimental studies in animal models and cell lines have demonstrated the additional roles of IL-1 in HBV infection. Ma et al. used an HBV mouse model to suggest that the IL-1R/Toll-like receptor (TLR) signaling pathway might contribute to HBV gene expression regulation and HBV clearance, since the deficiency of this pathway led to impaired CD8^+^ T-cell response and higher HBV replication [9]. Likewise, Li et al. showed that HBV can stimulate macrophages to undergo M1- or M2-like polarization, which in turn can suppress HBV gene expression and viral replication through IL-1b release [10]. Yang et al. also showed, in cell cultures, that IL-1 signaling, induced by hepatitis B envelope antigen (HBeAg), stimulates T-helper 2 (Th2) cell activation, enhancing the immune response to HBV and thus viral clearance [11]. This inhibitory effect of IL-1 was also shown in a study by Delphin et al., using HepaRG cells, a human hepatocytes cell line infected with HBV, and monocytes from healthy donors [91].

Similarly, Watashi et al. showed that IL-1 and tumor necrosis factor alpha (TNF-α) were able to reduce HBV infection susceptibility in HepaRG cells through the induction of activation-induced cytidine deaminase (AID) [92].

The significant role of IL-1 in HBV infection has also been shown in a number of human studies. In one of them, Ozeki et al. proved that IL-1b was higher in patients with CHB when compared with controls; in the same study, serum IL-1b levels were inversely correlated with the severity of the infection [93]. These findings seem to be in line with a study by Hu et al., coming 33 years later; in this study CHB patients and Huh 7 cells were treated with tenofovir disoproxil fumarate and/or pegylated-Interferon-alpha. Expression of IL-1b was positively correlated with a good antiviral response [94]. Likewise, in a study by Vukobrat-Bijedic et al., serum IL-1b levels were higher in patients with CHB when compared with controls; these levels were also higher in patients with detectable HBV DNA in peripheral blood [95]. Molyvdas et al. in a study comprising liver biopsy specimens from a total of 66 patients with either HBV or HCV infection, showed that CHB patients experienced higher IL-1b expression, which in turn was positively correlated with tissue inflammation and higher levels of serum transaminases [96]. Collectively, IL-1 seems to play an integral role in host defense against HBV, through viral replication suppression, enhancement of anti-viral immune responses and inhibition of viral entry in the hepatocytes. Since anti-IL-1 agents are commonly used in autoimmune diseases, physicians should always keep in mind HBV reactivation risk in patients treated with those agents.

## 3. Interleukin-2

Interleukin-2 (IL-2) plays an important role in immune system regulation, as it is involved in T-cell differentiation and regulates immune response and homeostasis. IL-2 stimulation is crucial for the maintenance of T-regulatory (Treg) cells and the differentiation of CD4+ cells into effector T-cell subsets following antigen-mediated activation [97]. In CD8+ cells, IL-2 signals promote effector T-cell generation and differentiation into memory cells [12]. Interestingly, the use of IL-2 can amplify CD8+ T-cell responses, whereas the application of neutralizing IL-2 specific antibodies induces the expansion of the Treg cell population, thus favoring either immune stimulation or suppression [12,13,14,15,98].

IL-2 has been studied in CHB, mainly due to its role in HBV-specific T-cells [15]. In one of the first studies assessing IL-2 activity in patients with CHB, Anastassakos et al. proved that mononuclear cells of patients with CHB showed significantly reduced activity of IL-2 compared with controls [99]. On the contrary, Ozeki et al. showed that serum IL-2 values in patients with CHB were higher than in controls and correlated with the degree of liver damage [93], a finding in line with the results of Debnath et al. [100].

Since IL-2 seems to lead to the production and proliferation of CD8+ cells, it has reasonably been proposed as a therapeutic option for CHB in a variety of trials. Onji et al. used recombinant IL-2 in 11 patients; only 2 achieved seroconversion [101]. Furthermore, in a larger study by Artillo et al. comprising 31 patients with CHB, IL-2 treatment failed to lead to significant seroconversion rates [102]. In a very interesting study using IL-2 as a treatment option for CHB, the EU-funded 2LIVEr project aims to reactivate the ineffective CD8+ T-cell response towards HBV through hepatocellular delivery of IL-2 by a lentiviral vector [103].

Overall, IL-2 seems to be an interesting treatment option for HBV infection, mainly due to the enhancement of anti-viral CD8+ responses.

## 4. Interleukin-4

Interleukin-4 (IL-4) is a crucial anti-inflammatory cytokine secreted mainly by Th2 cells that leads to proliferation, differentiation and ultimately antibody production by mature B-cells. It shifts the adaptive immune response toward humoral immunity, as it promotes naïve CD4+ cells to differentiate into Th2 cells and inhibits interferon gamma (IFN-γ) production as well as T-helper 1 (Th1) responses [104].

Li et al. demonstrated, in a study comprising 60 patients with CHB, a positive correlation between HBV viral load and serum levels of IL-4, with IL-4 serum levels progressively diminishing from the HBV immune-tolerant to HBV inactive-carrier and HBV immune-active phases [105]. On the contrary, Jiang et al., in a study with 22 patients with CHB under adefovir dipivoxil treatment, showed low levels of serum IL-4 upon treatment initiation and improvement after 104 weeks of treatment [106]. Likewise, Gu et al. [107] and Zhang et al. [108] reported a negative correlation between serum levels of IL-4 and HBV viral load, stating that these conflicting results may derive from gene polymorphisms.

Moreover, a handful of studies have demonstrated the capability of IL-4 to cease HBV replication in specific HCC cell lines [16,17].

As IL-4 boosts Th2 and inhibits Th1 immune responses, hence reducing liver inflammation, it could serve as a potential treatment option for HBV infection.

## 5. Interleukin-5

Interleukin-5 (IL-5) is a cytokine that augments humoral immune response through promotion of maturation, differentiation and survival of B-cells. It is primarily produced by Th2 cells, and secondarily by mast cells, eosinophils and natural killer T-cells (NKT) [18]. IL-5 plays a vital role in the immune system’s reaction against several infections [19,109,110], leading to leukocyte expansion and intensification of their activation status [111]. Recently published data show that IL-5 might play a pivotal role in HBV infection. Vimali et al. demonstrated in a study, comprising 30 patients with CHB, a negative association between serum levels of IL-5 and HBV DNA, indicating that IL-5 may contribute to HBV replication control [19]. In addition, Wang et al. showed that serum concentration of IL-5 after 24 weeks of therapy with a combination of nucleos(t)ide analogs (NAs) and pegylated interferon alpha (Peg-IFNα) was able to predict patients’ treatment response and the likelihood of achieving HBsAg seroclearance [112]. Finally, Badary et al. claimed that pretreatment levels of IL-5 in patients with CHB who received entecavir for a year were useful in predicting virologic response, further highlighting the close connection between IL-5 and HBV infection [113].

IL-5 seems to play an important role both in HBV suppression, as well as a predictive marker for treatment virological response; however, more data is required until safe consumptions are reached.

## 6. Interleukin-6

IL-6 is characterized by pleiotropic functions, involving not only immune response stimulation but also liver homeostasis and protein expression. IL-6 stimulates CD4+ T-cells, leading to antibody production by B-cells and enhancing the differentiation of naive T-cells to T-helper 17 (Th17) cells, while it also inhibits the differentiation of Tregs [20,21].

IL-6 also promotes liver regeneration and protects liver cells from injuries caused by immune responses, alcohol and viral infections [22,23,114].

Several studies have suggested that IL-6 plays a crucial role in HBV infection. Hosel et al. reported that IL-6, released from Kupffer cells, activates the mitogen-activated protein kinases exogenous signal-regulated kinase (ERK) 1/2 and c-jun N-terminal kinase (JNK), which down-regulate the expression of HNF-1a and 4a and thus negatively affect the entry of HBV into the hepatocyte in a dose-dependent way [24]. Moreover, the homodimerization of gp130, induced by IL-6 binding to its receptor, activates the Janus kinase (JAK)-signal transducer and activator of the transcription 3 (STAT3) signaling pathway. The interaction of this pathway with the HNF-3 complex and HBV enhancer 1 (Enh1) seems to control HBV-encoded oncogene X protein (HBx) expression and HBV replication [21].

Kuo et al. demonstrated that IL-6 effectively suppressed HBV replication in an HBV-producing cell line through viral transcripts and viral genome-containing nucleocapsids reduction, and thus suggested that IL-6 could prevent cccDNA accumulation [22].

As expected, the relationship between IL-6 and HBV has been investigated in multiple human studies. In the largest of them, comprising a total of 641 patients with CHB, Tang et al. showed that serum levels of IL-6 in patients with CHB correlated with the severity of infection, being higher in patients with cirrhosis or HCC when compared with inactive carriers and in chronic active than chronic persistent hepatitis, highlighting the critical role of IL-6 in HBV-related liver inflammation and regeneration [115]. Likewise, both Bekçibaşı et al. and Tangkijvanich et al. showed that IL-6 serum levels of patients with CHB were significantly higher than controls and correlated with disease severity [116,117]. Finally, Wu et al. claimed that IL-6 plasma levels could predict CHB progression to acute liver failure and they proposed its use as an early biomarker for HBV-related acute-on-chronic liver failure (HBV-ACLF) [118].

As explained, IL-6 seems to play a critical role in HBV infection. In this line, physicians should be aware of the possibility of HBV reactivation when treating patients with autoimmune diseases and concurrent HBV infection with anti-IL-6 drugs [119].

## 7. Interleukin-10

Interleukin-10 (IL-10) plays a unique role in the anti-inflammatory regulation of the immune system. Its main cellular sources are monocytes/macrophages, as well as Th1, Th2, Th17 and Tregs. IL-10 is, to a lesser extent, produced by B-cells, dendritic cells (DC) and NKT cells [25,26,27,120]. IL-10 diminishes the secretion of pro-inflammatory cytokines and mediators and regulates the expression of cell surface molecules by myeloid cell subsets. In addition, it inhibits antigen presentation, thus acting as a potent anti-inflammatory cytokine, inducing immune tolerance and viral persistence [25,26,27,28,29,120].

As anticipated, IL-10 plays a pivotal role in HBV infection; in CHB patients, higher levels of IL-10 have been consistently noted [28,30,31]. Specifically, IL-10 enhances HBV replication and suppresses HBV-specific CD8+ T-cell immune responses, providing a defensive mechanism to mitigate immune-mediated hepatic injury that ultimately results in an increase in viral load and HBV infection persistence [30,31,32,33,34,35,36]. The importance of IL-10 in alleviating hepatic injury is shown in a study by Wang et al., where, in patients with HBV-ACLF, a decrease in IL-10 serum levels after HBV exacerbation was noted [37].

The value of IL-10 in the outcome of HBV infection is also highlighted in a recent study by Rybicka et al., where the genetic variation within the IL-10 gene influences the chronicity of hepatitis B and virus-induced liver injury. In this study, IL-10 genetic variation is even associated with treatment-induced HBsAg seroclearance [121].

Although IL-10 is generally recognized as an immunosuppressive cytokine, a couple of studies have shown that its simultaneous expression with other cytokines, like IL-2 and interleukin-12 (IL-12), can significantly increase HBV-related cytotoxicity [122,123]. Moreover, Wu et al. showed that elevated levels of IL-10 and IL-12 in HBeAg-positive patients are associated with spontaneous HBeAg seroconversion at an early stage [123], while in two other studies, increased levels of IL-10 and IL-10/HBV DNA ratio were correlated with non-response to interferon alpha (IFN-α) treatment [124,125].

Notably, Shi et al. suggested that, in HBV-induced HCC, diminished levels of IL-10 within the tumor microenvironment are associated with decreased tumor size and up-regulation of HBV-specific IFN-γ-secreting tumor-infiltrating lymphocytes (TIL) [126].

Overall, IL-10 seems to be pivotal in HBV replication and suppression of the anti-viral immune response; however, its anti-inflammatory role could be protective against the possibility of viral-related hepatotoxicity leading to liver failure.

## 8. Interleukin-12

IL-12, primarily produced by DC and phagocytes, serves as an immunomodulatory and pro-inflammatory cytokine [38,39,40]. IL-12 stimulates IFN-γ production, regulates the differentiation of Th1 cells, activates NK cells, and enhances their proliferation and cytotoxicity. Consequently, it acts as a crucial bridge connecting innate and adaptive immunity [38,39,40]. IL-12 secretion by DC subsets in response to various pathogens is dependent on different regulation of genes encoding IL-12, TLR expression, and cross-regulation between the different DC subsets, involving cytokines such as IL-10 and type I IFN [38]. Moreover, IL-12 down-regulates Tregs through the stimulation of nitric oxide production by antigen presenting cells (APCs), like macrophages [41,127]. Due to its close relation with lymphocyte activation, IL-12 is considered a potent anticancer agent with a significant role in T-cell-mediated cytolysis of cancer cells and malignant antigen presentation [128,129].

Regarding HBV infection, IL-12 seems to enhance HBV-related cytotoxicity and replenish the exhausted HBV-specific CD8+ T-cells in persistent CHB [23,42]. IL-12 serum levels and hepatic expression seem to be upregulated in patients with CHB [130]; HB core antigen (HBcAg) as well as HBx protein have been described as causal factors of this upregulation in various experiments [130,131]. Intriguingly, in a study by Rossol et al. comprising 72 patients with CHB, IL-12 levels were higher in patients when compared with healthy controls and, notably, IL-12 levels increased even further in patients under interferon treatment who showed virological response when compared to non-responders [132]. On the contrary, Zhou et al., in a cohort of 142 patients with CHB, showed that IL-12 levels were positively correlated with disease progression and could indicate disease severity for different HBV-DNA loads [133]. Finally, Du et al. demonstrated that HBV-ACLF patients had higher IL-12 serum levels [134].

Due to its physiological actions, IL-12 has been used as an anti-viral drug for HBV. Cavanaugh et al. reported that treatment of HBV-infected mice with IL-12 interrupted HBV replication, probably through the induction of TNF-α and IFN α/β and γ [135]. Similarly, in patients with CHB, the use of IL-12 was correlated with significantly lower HBV DNA concentrations at the end of treatment, as well as during the 24 weeks of follow-up [136,137]. In a study by Rigopoulou et al., IL-12 was combined with lamivudine as a CHB treatment. Even though this combination regimen showed better anti-viral activity than lamivudine monotherapy, HBV DNA increased after lamivudine discontinuation [138]. Finally, Yang et al. suggested that increased serum IL-12 after 48 weeks of treatment with entecavir maleate contributed to an increased probability of HBeAg seroconversion [139].

## 9. Interleukin-17

The interleukin-17 (IL-17) family comprises six members (IL-17A to IL-17F) that mediate their biological functions by binding to receptors IL-17RA and IL-17RE, which form receptor complexes and initiate downstream signaling events in the IL-17 signaling pathway [140]. The most studied IL-17 family members are IL-17A and IL-17F. These interleukins promote their biological activities by binding to the heterodimeric receptor complex composed of IL-17RA and IL-17RC or by forming a ternary complex with IL-17RA and IL-17RC [43,140,141]. IL-17A induces several pro-inflammatory responses, mainly the production of TNF-α and β, IL-1β, IL-6 and other pro-inflammatory cytokines from Kupffer cells, DCs, hepatic stellate cells (HSC) and monocytes [23,43]. IL-17 seems to play a pivotal role in host defense against various fungal, bacterial and viral infections, including influenza, human immunodeficiency (HIV) and HCV viruses [142,143,144]. Moreover, IL-17 has an established role in many autoimmune diseases, especially ankylosing spondylitis, psoriasis and psoriatic arthritis; anti-IL-17 monoclonal antibodies are, in fact, broadly used in treatment of these diseases [145,146,147].

Even though IL-17 seems to play a significant role in viral infections, its contribution to the immune response against HBV infection is still ill-defined. Wang et al. showed that in a human HBV-infected cell line culture, levels of HBV DNA and HBsAg decreased upon treatment with IL-17. The opposite was observed when anti-IL-17 was administered [148]. On the other hand, there are indications that IL-17A levels are associated with CHB, cirrhosis and HCC [44,45]. The elevated serum levels of IL-17 (derived from Th17 cells) in patients with CHB and their correlation with alanine aminotranferase (ALT) levels and the extent of liver injury have been verified in several studies [149,150,151,152,153,154]. Finally, HBV persistence and hepatocellular damage in HBV-infected individuals have been associated with altered Tregs/Th17 and Th1/Th17 ratio [44,152,155].

Altogether, the role of IL-17 in HBV infection is still uncertain, even though IL-17 has been associated with HBV-related liver injury.

## 10. Interleukin-21

Interleukin-21 (IL-21) is an immunoregulatory cytokine—secreted predominantly by follicular helper T (Tfh), Th17 and NKT cells [156,157]—that contributes to immune system regulation in a pleiotropic manner depending on its microenvironmental conditions [46]. IL-21 mainly controls maturation, activation and proliferation of CD4+, CD8+ T-cells and B cells, having a pivotal role in many autoimmune and inflammatory diseases [47,48,49].

Various studies have investigated the role of IL-21 in CHB, showing that it affects HBV-specific CD8+ T-cells that play a key role in suppressing HBV progression [50,51,52,53]. In particular, IL-21 down-regulates the inhibitory factors programmed cell death protein 1 (PD-1) and T-cell immunoglobulin and mucin-domain containing-3 (TIM-3), enhancing the cytotoxic capacity and promoting the sustainability and proliferation of these HBV-targeting cells and thus accomplishing viral control [53]. In addition, IL-21 secretion by Tfh cells seems to induce HBsAg-specific antibody production [158,159] and suppress the replication of HBV indirectly by preventing IL-10 secretion [160]. On the other hand, as IL-21 mediates a robust response by the immune system, it induces inflammation, and in that way, it participates in the development of augmented liver injury that can lead to fibrogenesis and HBV-associated liver cirrhosis [161,162,163,164,165].

Given its therapeutic potential, IL-21 has been tried in mouse models, resulting in HBV clearance [53,166,167,168], while showing promising results even on the pre-exposure prevention of HBV [169]. Interestingly, Publicover et al., in another animal study, suggested that IL-21 could influence the outcome of HBV infection in an age-dependent manner. Young mice, due to their ineffective hepatic immune-priming environment, had decreased production of IL-21 that resulted in viral persistence; on the contrary, IL-21 sufficiency in adult mice led to HBV clearance [170].

Regarding human studies, Ma et al., in a study compromising 75 HBeAg-positive patients with CHB under telbivudine therapy, found that higher levels of serum IL-21 were associated with HBeAg seroconversion [171]. In addition, Huang et al. demonstrated that IL-21 concentrations at 0, 12, 24, 52 and 104 weeks after cessation of entecavir treatment were significantly lower in patients with virological relapse than in those in remission, suggesting that IL-21 could be used as a biomarker for HBeAg seroconversion [172]. Finally, Tang et al., in a recently published study, showed a brand-new mechanism for HBV clearance by combining IL-21 with antibodies against T-cell immunoreceptor with Ig and ITIM domains (anti-TIGIT), a combination that enhanced the antiviral efficacy of NK cells in CHB [173].

Overall, IL-21 seems to play a critical role in host defense against HBV infection by enhancing both humoral and adaptive immune responses. Interestingly, a handful of studies have shown that IL-21 could also serve as a biomarker for HBeAg seroconversion.

## 11. Interleukin-22

Interleukin-22 (IL-22), a member of the IL-10 family, controls tissue responses to inflammation. IL-22 is mainly secreted by T-helper 22 (Th22), Th17 and Th1 cells, as well as γδT cells, cytotoxic T-cell subsets and NKT cells. IL-22 targets and regulates tissue cells to protect them from damage and to induce their regeneration [54,55,56].

Current evidence presents IL-22 as a double-edge sword in HBV infection. Studies support that serum concentration of IL-22 is elevated in CHB [57,58,59,60], and so is the Th17 cells’ percentage in peripheral blood [58]. In mice, Feng et al. showed that CD3+ T-cells induce liver progenitor cell proliferation via IL-22 expression [61], while Park et al. demonstrated that IL-22 secretion may be a protective mechanism in preventing further liver injury, despite its positive association with serum ALT levels [62]. On the other hand, various studies have confirmed the pro-inflammatory role of this cytokine in HBV infection and have highlighted its correlation with disease progression and the grade of intrahepatic inflammation [57,58,60,63,64]. Recently, Zhang et al., using Th22 cells from healthy donors, and Shi et al., using Th22 cells from patients with CHB, atypical hyperplasia of the liver and HCC, demonstrated that the up-regulation of IL-22 in HBV infection is related to HCC progression [65,66], while Schwarzkopf et al., revealed that high IL-22 serum levels are associated with ACLF and mortality of cirrhotic patients [67]. Finally, Okuhara et al., in a study comprising 48 patients with CHB, showed that elevated IL-22 before treatment was correlated with a higher likelihood of virological response, further complicating the role of IL-22 in CHB [174]. A possible explanation for these contradictions is that the effect of IL-22 in the context of HBV infection might differ depending on the specific disease stage [68,69].

As far as the role of IL-22 as a treatment option in CHB is concerned, Wang et al. showed that IL-22 producing CD3+ CD8-T-cells were suppressed after 48 weeks of treatment with peg-interferon [57]. In addition, Hao et al. found a notable decrease in IL-22 levels after 48 weeks of telbivudine treatment in a cohort of 24 CHB patients [175], suggesting that IL-22 could be used as a biomarker for disease response to treatment.

Collectively, the exact role of IL-22 in HBV infection is still unknown, since IL-22 seems to exhibit both pro- and anti-inflammatory effects, as already shown in other diseases [176].

## 12. Interleukin 23

Interleukin-23 (IL-23) is a heterodimeric cytokine belonging to the IL-12 cytokine family. The main biological functions of IL-23 consist of promoting CD4+ T-cell proliferation and inducing IFN-γ and IL-12 production, thus enhancing DC antigen presentation [23,70,71].

Various studies have pinpointed the critical role of IL-23 in CHB, since IL-23 seems to be a potent mediator of HBV-related hepatic inflammation. Three at least HBV proteins—HBsAg, HBcAg and HBx—are capable of inducing IL-23 production, leading to differentiation of naive CD4+ T-cells into Th17-cells and enhancement of Th17-cell-mediated liver inflammation and fibrosis [72]. The importance of IL-23 in CHB is highlighted in a study by Xia et al., comprising 192 CHB patients, with 60 of them undergoing liver biopsy [73]. The authors showed that serum levels of IL-23 and hepatic IL-23 expression were positively correlated with serum HBV DNA, aspartate aminotransferase (AST) and ALT levels, while hepatic inflammation was positively correlated with IL-23 p19 expression [73]. In accordance with that, Wang et al. found higher IL-23/IL-17 pathway-related cytokines in liver tissue of patients with CHB when compared with those of healthy controls, while, in patients with acute on chronic HBV-related liver failure, high levels of IL-23 were strongly correlated with disease severity [74,75,76]. Finally, Li et al. supported that, in CHB, CD161+CD4+ T-cells play a pro-inflammatory role and facilitate liver fibrogenesis through an IL-23/IL-17 axis [177].

As opposed to other cytokines, like IL-22, the role of IL-23 in HBV infection seems to be quite straightforward; IL-23 production is enhanced by various HBV proteins and its overproduction leads to severe hepatic inflammation and consequent necrosis.

## 13. Interleukin 35

Interleukin-35 (IL-35) is a relatively new identified member of the IL-12 family, primarily secreted by Treg and regulatory B-cells (Breg) [178,179]. Its predominant action is immunosuppression, which is achieved through the inhibition of T-cell proliferation and effector functions [77,180]. IL-35 has a crucial role in immune-related diseases and in the maintenance of immunological tolerance [178,181,182,183,184]. Given the crucial role of Treg in blocking effective immune responses against HBV, IL-35 could be a very important contributor in CHB and even used as a therapeutic target [185]. Moreover, IL-35 seems to play a pivotal role in regulating the virus-specific Treg/Th17 balance [78]. The latter function constitutes this interleukin, an active contributor in the control of liver inflammation, by dampening antiviral immune responses and thus suppressing inflammatory responses [79,80,81].

As a matter of fact, a growing number of studies have shown the importance of IL-35 in HBV infection. Tao et al. showed, using the HepG2-NTCP HBV infection cell model, that IL-35 could promote HBV replication; IL-35 treatment led to higher levels of HBV DNA, even though no differences in serum transaminases were noted between the treated group and controls [82]. Likewise, patients with CHB seem to have higher serum levels of IL-35 when compared with controls, with IL-35 being positively correlated with HBV DNA levels [82,83,84,85,86,87,88], while it also seems to suppress the proliferation of HBV antigen-specific CTLs and interferon (IFN)-*γ* production both in vitro and ex vivo [89,90]. Interestingly enough, in all of these studies, liver inflammation was largely dampened, accessed either by serum transaminases or liver biopsies, highlighting the anti-inflammatory effect of IL-35.

Taken together, the above data support the multifaceted emerging effect of IL-35 on viral persistence and immune tolerance during HBV infection. Future investigation is essential for an in-depth understanding of IL-35′s immune regulation mechanisms, which may yield new immunotherapeutic strategies.

## 14. Conclusions

In summary, most ILs play a critical role in HBV life cycle as well as in consequent liver inflammation. Among them, the pro-inflammatory ILs, namely IL-1, IL-5, IL-6, IL-12 and IL-21, seem to suppress HBV replication both directly and through immune system activation, while the anti-inflammatory ILs, namely IL-10, IL-23 and IL-35, act more probably as HBV replication enhancers. Interestingly enough, IL-17 has been closely related to HBV-induced liver injury, even though its exact role in the clinical course of HBV infection is under debate. Likewise, the role of IL-22 is largely ill defined, even though preliminary data show that its role in HBV replication proliferation and subsequent liver damage could be extremely important.

## Figures and Tables

**Table 1:** Interleukins presented in the text with their principal effects in immune response and their role in HBV infection.

Interleukins	Principal Effects	Contributions in HBV Infection	References
IL-1	Proinflammatory	Inhibition of HBV entry and replication Th2-cell activation	[7,8,9,10,11]
IL-2	Promotion of effector T-cell differentiation Maintenance of Tregs for suppressive functions	Regulation of HBV-specific T-cells	[12,13,14,15]
IL-4	Promotion of Th2-cell differentiation (humoral immunity)	Suppression of Th1-cell response Cessation of HBV replication in specific HCC cell lines	[16,17]
IL-5	Maturation, differentiation and survival of B-cells	Probable negative association with HBV replication	[18,19]
IL-6	Stimulation of CD4+ T, B and Th17-cells Inhibition of Tregs	Inhibition of HBV entry and replication	[20,21,22,23,24]
IL-10	Anti-inflammatory	Enhancement of HBV replication Suppression of HBV-specific T-cell response Mitigation of hepatic injury	[25,26,27,28,29,30,31,32,33,34,35,36,37]
IL-12	Proinflammatory	Enhancement of HBV-specific CD8+ T-cells in CHB Downregulation of Tregs	[23,38,39,40,41,42]
IL-17	Proinflammatory	Ill-defined role in HBV infection Probable association with CHB, cirrhosis and HCC	[23,43,44,45]
IL-21	Pleiotropic-Immunoregulatory	Boost of HBV-specific CD8+ T-cells and HBV suppression	[46,47,48,49,50,51,52,53]
IL-22	Promotion of cellular proliferation, resistance to apoptosis and tissue regeneration Pro-inflammatory	Regulation of intrahepatic inflammation; tissue protection or liver injury progression depending on disease stage	[54,55,56,57,58,59,60,61,62,63,64,65,66,67,68,69]
IL-23	CD4+ T-cell proliferation Enhancement of DC antigen presentation	Regulation of HBV-related hepatic inflammation	[23,70,71,72,73,74,75,76]
IL-35	Anti-inflammatoryImmunosuppression	Stimulation of HBV replication Dampening of cytolytic and non-cytolytic activity of CTLs	[77,78,79,80,81,82,83,84,85,86,87,88,89,90]

Abbreviations: IL: Interleukin, HBV: Hepatitis B virus, Th2-cells: T helper cells type 2, Tregs: Regulatory T-cells, Th1-cells: T helper cells type 1, HCC: Hepatocellular carcinoma, Th17-cells: T helper cells type 17, CHB: chronic hepatitis B, DC: dendritic cell, CTLs: Cytotoxic T-cells.

## Data Availability

No new data were created or analyzed in this study. Data sharing is not applicable to this article.
